# Highly crystalline CsPbI_2_Br films for efficient perovskite solar cells *via* compositional engineering

**DOI:** 10.1039/c9ra06363c

**Published:** 2019-09-26

**Authors:** Fang He, Wenzhan Xu, Meng Zhang, Xuan Zhang, Baofu Ding, Guodan Wei, Feiyu Kang

**Affiliations:** Tsinghua-Berkeley Shenzhen Institute (TBSI), Tsinghua University Shenzhen 518000 China weiguodan@sz.tsinghua.edu.cn fykang@sz.tsinghua.edu.cn; Tsinghua Shenzhen International Graduate School, Tsinghua University Shenzhen 518000 China; School of Materials Science and Engineering, Tsinghua University Beijing 100084 China

## Abstract

All-inorganic CsPbI_2_Br shows high thermal stability for promising application in perovskite solar cells (PSCs). The performance of PSCs is significantly affected by their morphology and crystallinity induced by compositional ratio, solvent/anti-solvent engineering and post thermal annealing. In this study, the compositional ratio effect of two precursors, PbI_2_ and CsBr, on the power conversion efficiency (PCE) of a device with ITO/SnO_2_/CsPbI_2_Br/Spiro-MeOTAD/Au structure was investigated. With the assistance of anti-solvent chlorobenzene, perovskite with a PbI_2_ : CsBr ratio of 1.05 : 1 showed a high quality thin film with higher crystallinity and larger grain size. In addition, the molar ratio of precursors PbI_2_ and CsBr improved the PCE of the PSCs, and the PSCs fabricated using the perovskite with an optimal ratio of PbI_2_ and CsBr exhibited a PCE of 13.34%.

## Abstract 2

All-inorganic CsPbI_2_Br shows high thermal stability for promising application in perovskite solar cells (PSCs).

## Graphical abstract

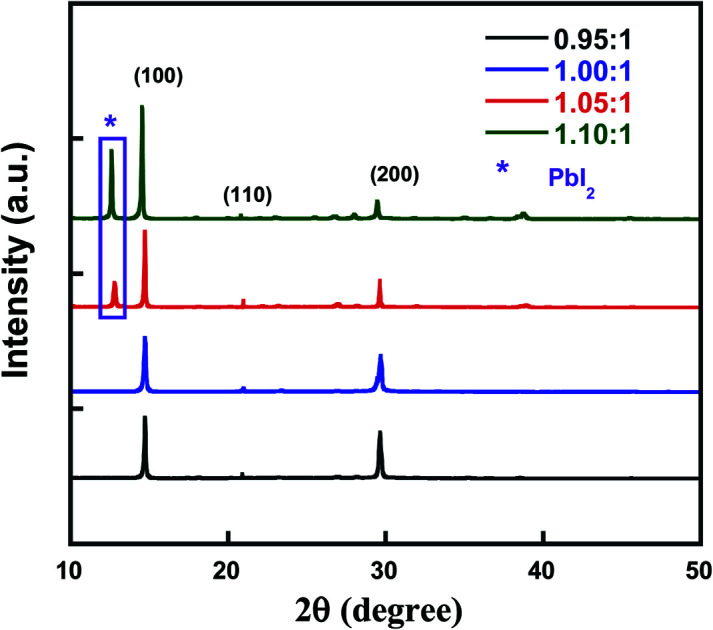

## 1. Introduction

Due to its appropriate optical band gap, high optical absorption coefficient, good ion migration ability and long carrier life, organic–inorganic hybrid perovskite is considered as an ideal material for use in the light absorption layer of solar cells.^1–5^ In the past decade, new breakthroughs have been made in the research of perovskite solar cells (PSCs).^6–8^ So far, the highest power conversion efficiency (PCE) of organic–inorganic hybrid perovskite solar cells has reached 25.2%,^9^ which is higher than the efficiency of commercial silicon solar cells. However, the poor long-term stability seriously limits the large-scale commercial use of organic–inorganic hybrid perovskite solar cells.^10–12^ Although some studies have effectively improved the stability of hybrid perovskite solar cells,^13,14^ they still cannot meet the requirements for practical applications. But the all-inorganic halide perovskite with good thermal stability can be obtained by completely replacing the organic components (MA^+^, FA^+^) of mixed perovskite with Cs^+^.^15^

Typical all-inorganic halide perovskite materials mainly include CsPbI_3_,^16^ CsPbI_2_Br,^17^ CsPbBr_3_ (ref. 18) and CsSnI_3_.^19^ Among them, CsPbI_3_ and CsPbI_2_Br of cubic phase have appropriate optical band gaps, 1.73 eV and 1.92 eV, respectively.^16,20^ Therefore, these two halide perovskites have been extensively studied and the PCE of devices has been rapidly improved.^20^ Nevertheless, there is still a big gap between the current maximum efficiency and the theoretical schottky limit,^20^ which suggests that the all-inorganic perovskite solar cells still have great room for improvement and research value in photoelectric conversion efficiency. On the other hand, the all-inorganic halide perovskite usually suffers a severe phase transition problem. In ambient air environment, CsPbI_3_ and CsPbI_2_Br of cubic phase are easily converted into non-perovskite phase, which will seriously affect their photoelectric performance and practical utilization of devices.^21^

Studies have shown that, compared with CsPbI_3_, CsPbI_2_Br is easier to maintain the cubic phase structure, which has intrigued great attention.^22^ Chen *et al.* confirmed that the a-phase and b-phase CsPbI_2_Br have better thermal stability and phase stability than CsPbI_3_ due to the mixing of halide elements through theoretical calculations.^23^ Through interface engineering,^24^ solvent engineering,^25^ precursor engineering^26^ and other strategies, the photoelectric performance of CsPbI_2_Br has been effectively improved. For example, Guo *et al.*^27^ successfully synthesized Rb-doped CsPbI_2_Br film with improved crystallinity and light absorption. Based on this, the prepared all-inorganic perovskite solar cells without hole transport layer achieved stabilized PCE of over 11%. Bai *et al.*^28^ improved the crystallinity of the CsPbBrI_2_ film by preheating the precursor solution at 100 °C, and reached a high efficiency of up to 14.81% with the device structure of FTO/TiO_2_/CsPbBrI_2_/CsPbBrI_2_ QDs/PTAA/Au. Chen *et al.*^29^ precisely controlled the growth of α-CsPbI_2_Br crystal by the synergistic effect of gradient thermal annealing and anti-solvent method, and obtained a high-quality film with few defects and good stability, achieving a record PCE of 16.07%. Liu *et al.*^30^ successfully designed a novel structure of solar cell by using ZnO@C_60_ bilayer as the electron transport layer, achieving a high PCE of 13.3% with good thermal stability. Dong *et al.*^31^ used EtOH anti-solvent and optimized CsBr deposition cycle to obtain a high-purity phase and high-quality CsPbI_2_Br film, successfully achieving a record PCE of 10.21% for the α-CsPbI_2_Br PSCs without hole transporting material.

Herein, we studied the effect of raw material stoichiometric ratio on the properties of CsPbI_2_Br. Four perovskite solar cells with ITO/SnO_2_/CsPbI_2_Br/Spiro-MeOTAD/Au were fabricated by changing the molar ratios of PbI_2_ and CsBr (0.95 : 1, 1.00 : 1, 1.05 : 1, 1.10 : 1). We found that excessive PbI_2_ would lead to obvious PbI_2_ peak in the XRD pattern, and a small amount of PbI_2_ would significantly affect the morphology and crystallinity of as-prepared CsPbI_2_Br perovskite films. In addition, different proportions of PbI_2_ and CsBr will also lead to changes in optical band gap and light absorption properties of perovskite thin films, as well as differences in CsPbI_2_Br phase stability. Our results show that solar cell devices have the best power conversion efficiency when the molar ratio of PbI_2_ and CsBr is 1.05 : 1. The enhanced PCE originates from the higher crystallinity and defects-free of perovskite thin film with appropriate grain size characterized by X-ray diffraction (XRD) and can electronic microscopy (SEM).

## 2. Experimental

### 2.1 Materials

Lead iodide (PbI_2_, 99.999%) and tin(iv) oxide (SnO_2_) colloid precursor were purchased from Alfa Aesar; cesium bromide (CsBr, 99.999%), chlorobenzene (CB, 99.8%), anhydrous dimethyl sulfoxide (DMSO, ≥99.9%), *N*,*N*-dimethylformamide (DMF, 99.8%), 4-*tert*-butylpyridine (*t*BP), tris(2-(1*H*-pyrazol-1-yl)-4-*tert*-butylpyridine)cobalt(iii)-tris(bis(trifluoromethylsulfonyl) imide) (FK209) and bis(trifluoromethylsulfonyl)amine lithium salt (Li-TFSI) were purchased from Sigma Aldrich. Anhydrous methanol, ethanol (>99.5%) and isopropyl alcohol (IPA, 99.5%) were purchased from Acros; Spiro-MeOTAD (99.8%) was purchased from Xi'an Polymer Light Technology Corp. All materials and reagents were used as received without further purification.

### 2.2 Precursor preparation

1 M CsBr and *X* M PbI_2_ (*X* = 0.95, 1.00, 1.05, 1.10) were dissolved in a mixed solvent (DMSO : DMF = 9 : 1) according to certain stoichiometric ratios, and stirred at 70 °C for 12 h. The CsPbI_2_Br precursor solution was then obtained by filtration through a 0.22 μm PTFE filter.

### 2.3 Solar cells fabrication

The ITO glasses were successively cleaned with acetone, ethanol and deionized water respectively by ultrasonic cleaning for 15 minutes, respectively. Before the spin coating, each ITO glass was blown dry with nitrogen gun and cleaned with plasma for 60 s. SnO_2_ was selected as the electron transport layer. We diluted the SnO_2_ colloid precursor to 5% with deionized water, then spin-coated at 3000 rpm for 30 s, and thermal annealed at 150 °C for 30 min to obtain an electron transport layer. Then the 1 M CsPbI_2_Br precursor solution was deposited on the SnO_2_ layer at the speed of 1000 rpm for 10 s and 4500 rpm for 35 s in a glove box, annealing at 260 °C for 10 minutes. For deposition of hole-transporting material, Spiro-MeOTAD was dissolved in chlorobenzene with a concentration of 80 mg mL^−1^, then 35 μL of lithium bis (trifluoromethanesulfonyl)imide in acetonitrile (260 mg mL^−1^) and 30 μL of 4-*tert*-butylpyridine was added into the Spiro-MeOTAD solution. The mixture was coated onto the perovskite film at 3500 RPM for 30 s to form a Spiro-MeOTAD hole-transporting layer. Finally, the counter electrode was deposited by thermal evaporation of 80 nm-thick gold under a pressure of 2 × 10^−6^ mbar. The active area was measured to be 0.045 cm^2^.

### 2.4 Characterization of perovskite thin films

UV-vis absorption and photoluminescence (PL) spectra of thin films were recorded on a HP 8453 spectrophotometer and FLS920 spectrofluorimeter (Edinburgh Instruments), respectively. A 150 W, ozone-free xenon arc lamp was used in PL measurements. Scanning electron microscope (SEM) images were obtained by using a field emission scanning electron microscope (JEOL-7401). Thicknesses of thin films were measured by Dektak 150 surface profilometer. X-Ray Diffraction (XRD) patterns were measured by an X-ray diffractometer Bruker D8 Advance using Cu Kα radiation source with a scan rate of 10° min^−1^.

### 2.5 Characterization of perovskite solar cells

The *J*–*V* characteristics of PSCs were measured by a Keithley model 2400 source measure unit (Newport, Oriel AM 1.5 G, 100 mW cm^−2^). The light intensity of 100 mW cm^−2^ was calibrated by a silicon reference cell. The external quantum efficiency (EQE) spectra of PSCs were performed on a DSR100UV-B spectrometer with a bromine tungsten light source, a SR830 lock-in amplifier and a calibrated Si detector.

## 3. Results and discussion

**Fig. 1:**
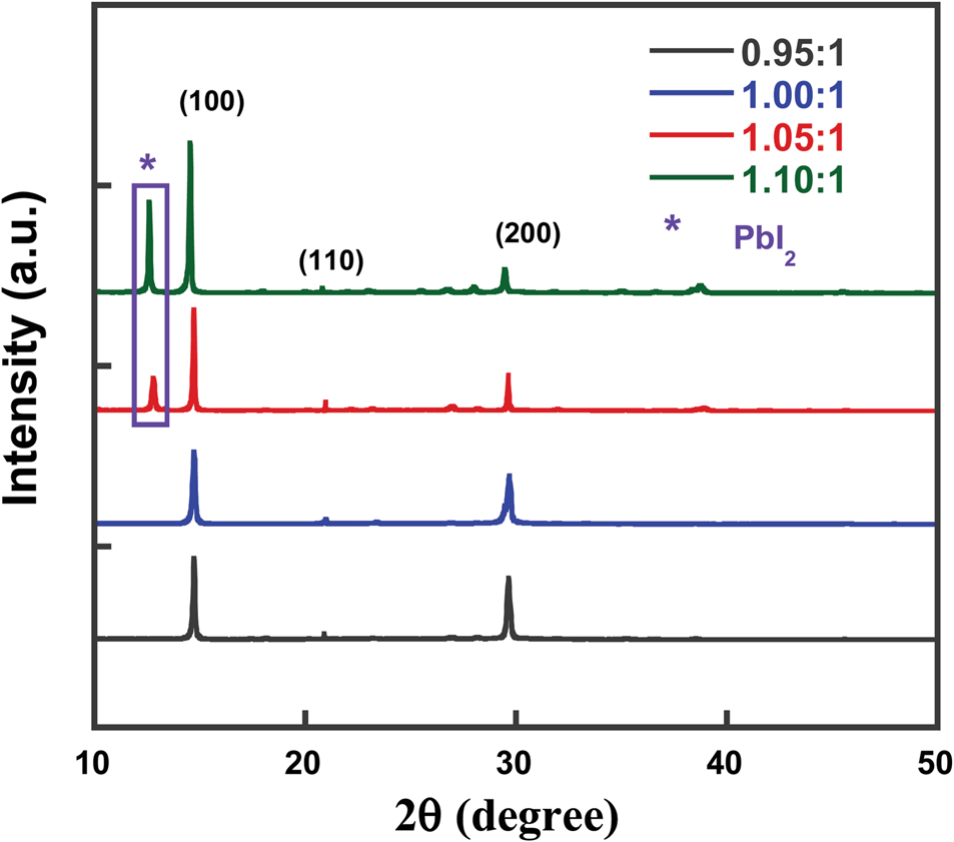
The XRD patterns of perovskite thin films with different molar ratio of PbI_2_ : CsBr.

To investigate the influence of compositional ratio of PbI_2_ : CsBr on the structural evolution of all-inorganic perovskite, X-ray diffraction (XRD) of these thin films are conducted in Fig. 1. Similar to perovskite with PbI_2_ : CsBr of 1.00 : 1, all other perovskite with PbI_2_ : CsBr (0.95 : 1, 1.05 : 1 and 1.10 : 1) thin films exhibited peaks at 14.7°, 20.9° and 29.6°, which correspond to the (100), (110) and (200) crystal planes of CsPbI_2_Br perovskite thin film, respectively, demonstrating prepared perovskites retain cubic phase structure, which is favorable for reducing charge recombination and promoting device performance.^26,32^ The corresponding average grain size were 61.34, 61.25, 53.75 and 55.83 nm calculated based on Scherrer formula, indicating the small adjusted component ratio of the PbI_2_ added into the precursor solution doesn't change crystal size significantly which is beneficial to improve the charge transport in the solar cells. The new peak located at 12.5° detected from both perovskite with PbI_2_ : CsBr (1.05 : 1 and 1.10 : 1) is likely to due to the peak of excess unreacted PbI_2_. It was reported that moderate excess PbI_2_ can functioned as role of passivating the surface defects for reducing the charge carrier recombination.

**Fig. 2:**
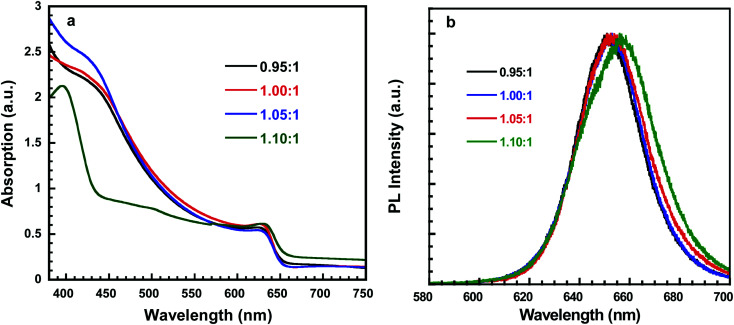
(a) Absorption of perovskite thin films with different molar ratio of PbI_2_ : CsBr. (b) Photoluminescence (PL) spectra of perovskite thin films with different molar ratios of PbI_2_ : CsBr.

Fig. 2a showed the absorption spectrum of perovskite thin films, all of which exhibited the typical absorption profile for cubic phase CsPbI_2_Br thin film with the typical absorption peak of 625 nm, which is consistent with other report.^31,33^ However, the perovskite film deposited with PbI_2_ : CsBr ratio of 1.10 : 1 has the absorption peak at 635 nm. The slightly red shifted absorption peak indicates the excess PbI_2_ actually influences the CsPbI_2_Br perovskite thin films. In addition, the peak is observed at 405 nm for the δ-perovskite with PbI_2_ : CsBr of 1.10 : 1, implying that the black α-CsPbI_2_Br phase could have been partially transitioned into the yellow δ-CsPbI_2_Br phase. Fig. 2b depicted the steady-state photoluminescence (PL) spectra of these four perovskite thin films. The emission peak of the CsPbI_2_Br thin film is at 653 nm is well consistent with absorption edges for corresponding perovskite thin films (Fig. 2a). It is also observed that a red shifted PL peak for perovskites with the molar ratio of 1.10 : 1.

**Fig. 3:**
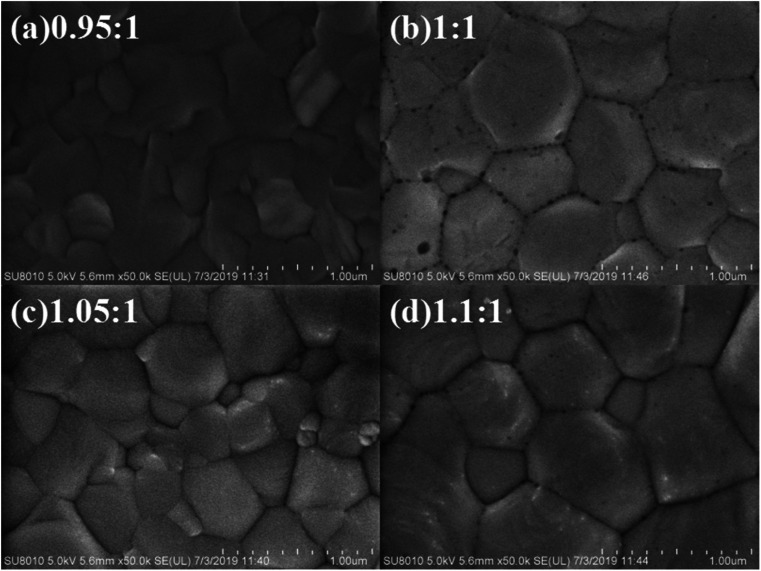
(a) The top-view SEM image of CsPbI_2_Br thin film with molar ratio of PbI_2_ : CsBr of (a) 0.95 : 1, (b) 1.00 : 1, (c) 1.05 : 1 and (d) 1.10 : 1.

The surface morphology and crystalline size of CsPbI_2_Br thin films deposited with these four different molar ratios of PbI_2_ : CsBr is further analyzed by SEM images, as shown in Fig. 3. It is obviously that all samples display densely stacked grains on their surfaces which is consistently with the calculated average crystal size in nanoscale range. However, brighter dots on the surface of the perovskite grains are clearly observed when the molar ratio of PbI_2_ : CsBr is 1.10 : 1 (Fig. 3d), proofing the existence of the unreacted PbI_2_ particles could affect the thin film quality.

**Fig. 4:**
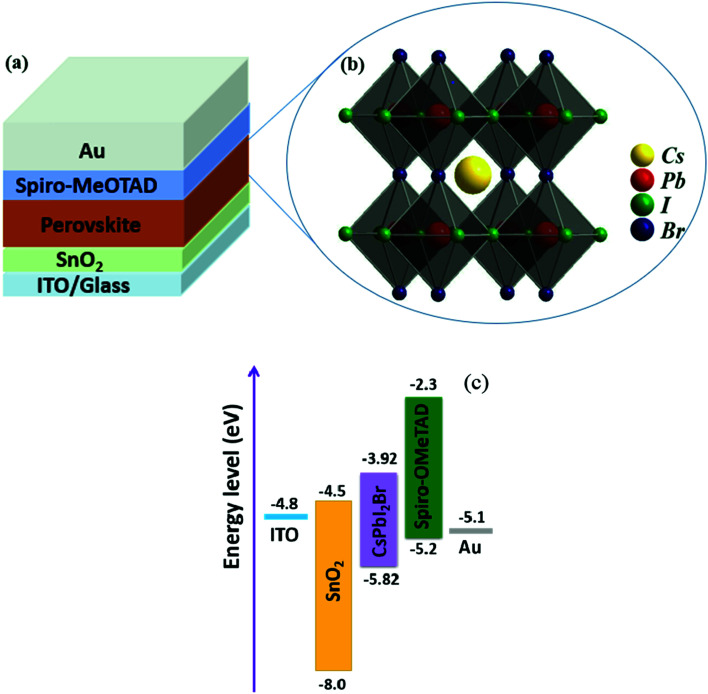
(a) Device structure of the PSCs. (b) Crystal structure of the perovskite CsPbI_2_Br. (c) the schematic energy diagram of glass/ITO/SnO_2_/perovskite/Spiro-OMeTAD/Au device.

**Fig. 5:**
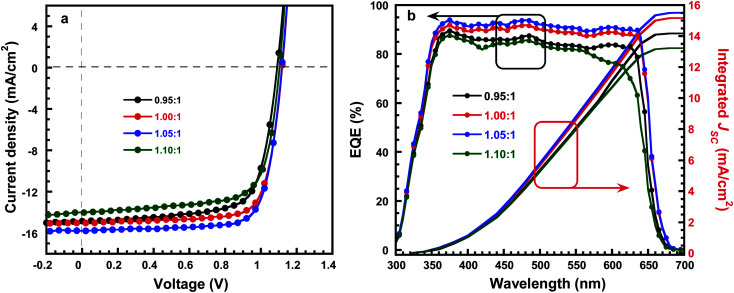
(a) *J*–*V* characteristics of CsPbI_2_Br thin film with molar ratio of PbI_2_ : CsBr. (b) EQE spectrum and integrated *J*_SC_ of CsPbI_2_Br thin film with molar ratio of PbI_2_ : CsBr.

To investigate the effect of composition ratios of PbI_2_ : CsBr on the photovoltaic performance of PSCs, devices with a planar heterojunction structure of ITO/SnO_2_/CsPbI_2_Br/Spiro-MeOTAD/Au (Fig. 4a) with the CsPbI_2_Br shown in Fig. 4b were fabricated where ITO acts as the cathode electrode, solution-processed SnO_2_ thin film is used as the electron extraction layer, Spiro-MeOTAD acts as the hole extraction layer and Au acts as the anode electrode, respectively (Fig. 4c). The current densities *versus* voltages (*J*–*V*) characteristics of PSCs under one-sun illumination with the light intensity of 100 mW cm^−2^, at the scan rate of 0.20 V s^−1^ and the reverse scan direction, are shown in Fig. 5a. The device performance parameters are summarized in Table 1. The PSCs based on the perovskite with PbI_2_ : CsBr of 0.95 : 1 and 1.00 : 1 show PCE of 11.49% (*V*_OC_ of 1.09 V, *J*_SC_ of 14.8 mA cm^−2^ and FF of 71.2%) and 12.52% (*V*_OC_ of 1.12 V, *J*_SC_ of 15.01 mA cm^−2^ and FF of 74.5%). The PSCs based on the perovskite with PbI_2_ : CsBr of 1.05 : 1 shows an enhanced performance upon slightly increasing with PbI_2_. Specifically, the PCE is increased to 13.34% with a *V*_OC_, *J*_SC_, and FF of 1.12 V, 15.78 mA cm^−2^, and 75.5%, respectively. It is worth noted that the simultaneously enhanced *V*_OC_, *J*_SC_ and FF is achieved. These enhancements are attributed to the denser, higher crystallinity CsPbI_2_Br grains which could effectively suppress charge recombination (Fig. 3c). However, further addition of much more PbI_2_ causes a negative performance change. For example, when the molar ratio of PbI_2_ : CsBr is increased to 1.10 : 1, the PCE is decreased to 10.91% (*V*_OC_ of 1.10 V, *J*_SC_ of 14.01 mA cm^−2^, FF of 70.8%). The excess PbI_2_ has reduced the sunlight absorption efficiency (Fig. 2a) and could act as recombination or trap centers with unnecessary spots on the active CsPbI_2_Br thin film (Fig. 3d), severally reducing the photocurrent and FF (Table 1). The strong correlation between *J*_SC_ and series resistance (*R*_s_) could be validated that with more and more PbI_2_ added, the measured *R*_s_ has consistently reduced when the molar ratio changes from 0.95 to 1.05 (Table 1), indicating the improved crystallinity does increase the charge separation and carrier transport efficiency. Thus the molar ratio of PbI_2_ : CsBr in the precursor solution is a crucial parameter for determining device performance.

**Table 1:** Performance parameters of PCSs with different molar ratio of PbI_2_ : CsBr

PbI_2_ : CsBr (molar ratio)	*V*_OC_ (V)	*J*_SC_ (mA cm^−2^)	FF (%)	PCE (%) (Best)	PCE (%) (Average)	*R*_s_ (Ω cm^2^)
0.95 : 1	1.09	14.80	71.2	11.49	10.43	53.26
1.00 : 1	1.12	15.01	74.5	12.52	11.85	43.87
1.05 : 1	1.12	15.78	75.5	13.34	12.78	35.55
1.10 : 1	1.10	14.01	70.8	10.91	9.85	48.12

EQE of all the perovskite solar cells is shown in Fig. 5b. The perovskite solar cells exhibit a photoresponse in the wavelength ranging from 300 to 650 nm, suggesting absorbed photos being converted into charge carriers, and further generates into photocurrent in the solar cells. In addition, the champion device shows a maximum value of about 93.4% at *λ* = 480 nm, the integrated *J*_SC_ values from the EQE spectra are 14.17 mA cm^−2^, 15.16 mA cm^−2^, 15.51 and 13.22 mA cm^−2^ for the PSCs fabricated by perovskite with PbI_2_ : CsBr of 0.95 : 1, 1.00 : 1, 1.05 : 1 and 1.10 : 1, respectively, which is about 5% error between the *J*_SC_ from EQE spectra and *J*–*V* curve, implying the accuracy of device performance.

**Fig. 6:**
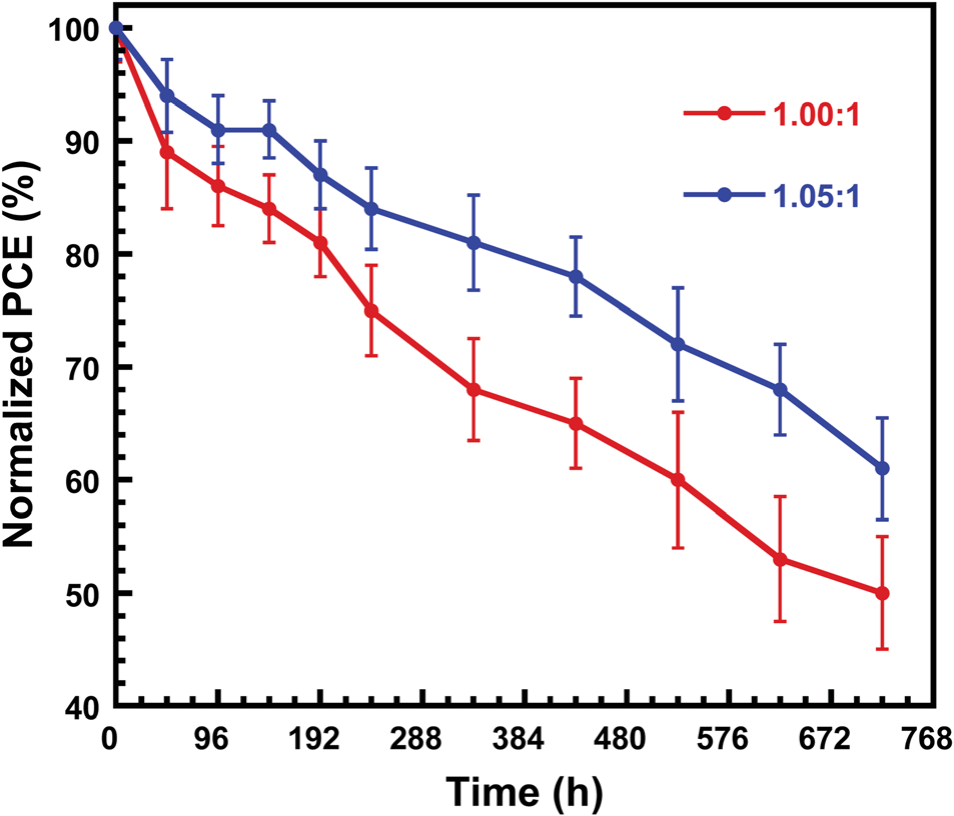
Normalized PCEs of the unencapsulated PSCs fabricated by perovskite with PbI_2_ : CsBr of 1.00 : 1 and 1.05 : 1 functioned as the time under in N_2_-filled glove box.

Furthermore, as the stability is the priority concern for the PSCs, the shelf stability of the PSCs is investigated. The devices are stored in N_2_ atmosphere without any encapsulation. As shown in Fig. 6, the PSCs fabricated by perovskite with PbI_2_ : CsBr of 1.05 : 1 showed a better shelf stability than that of PSCs fabricated by perovskite with PbI_2_ : CsBr of 1.00 : 1. After being stored for 240 h, PSCs fabricated by perovskite with PbI_2_ : CsBr of 1.05 : 1 retained 84% of the initial PCE, while the PCE of PSCs fabricated by perovskite with 1 : 1 ratio of the two precursors dropped to 75% of the initial value in the same storage condition and time. The enhanced shelf stability which we attribute to the surface passivation of unreacted PbI_2_.^34–36^

## 4. Conclusions

In summary, we investigated the effect of compositional engineering on the film properties and device performance of inorganic perovskite CsPbI_2_Br by changing the stoichiometric ratios of PbI_2_ and CsBr (0.95 : 1, 1.00 : 1, 1.05 : 1 and 1.10 : 1). The results show that the molar ratio of the raw materials is 1.05 : 1, the CsPbI_2_Br film shows the best crystallinity and the corresponding solar cell (ITO/SnO_2_/CsPbI_2_Br/Spiro-MeOTAD/Au) achieves the highest efficiency of 13.34%. XRD and SEM results show that once the molar ratio of raw materials is increased to 1.10 : 1, the resultant film contains more PbI_2_, which may provide more recombination centers for free electrons and holes, thus significantly reducing the efficiency of the device. Our study highlights the importance of compositional engineering and offers a meaningful reference for the preparation of stable and efficient solar cell devices.

## Conflicts of interest

There are no conflicts of interest to declare.

## Supplementary Material
